# Life Cells for future energy systems: adaptation, evolution and exploration

**DOI:** 10.1093/nsr/nwag065

**Published:** 2026-02-06

**Authors:** Jingning Lai, Fengling Zhang, Li Li, Feng Wu, Renjie Chen

**Affiliations:** Beijing Key Laboratory of Environmental Science and Engineering, School of Materials Science and Engineering, Beijing Institute of Technology, Beijing 100081, China; Beijing Key Laboratory of Environmental Science and Engineering, School of Materials Science and Engineering, Beijing Institute of Technology, Beijing 100081, China; Beijing Key Laboratory of Environmental Science and Engineering, School of Materials Science and Engineering, Beijing Institute of Technology, Beijing 100081, China; Innovative Research Team in High-Safety Energy Storage System and Smart Microgrids of Guangdong Province, Beijing Institute of Technology (Zhuhai), Zhuhai 519088, China; Shandong Key Laboratory of Advanced Chemical Energy Storage and Intelligent Safety, Advanced Technology Research Institute, Beijing Institute of Technology, Jinan 250300, China; Collaborative Innovation Center of Electric Vehicles in Beijing, Beijing 100081, China; Beijing Key Laboratory of Environmental Science and Engineering, School of Materials Science and Engineering, Beijing Institute of Technology, Beijing 100081, China; Innovative Research Team in High-Safety Energy Storage System and Smart Microgrids of Guangdong Province, Beijing Institute of Technology (Zhuhai), Zhuhai 519088, China; Shandong Key Laboratory of Advanced Chemical Energy Storage and Intelligent Safety, Advanced Technology Research Institute, Beijing Institute of Technology, Jinan 250300, China; Collaborative Innovation Center of Electric Vehicles in Beijing, Beijing 100081, China; Beijing Key Laboratory of Environmental Science and Engineering, School of Materials Science and Engineering, Beijing Institute of Technology, Beijing 100081, China; Innovative Research Team in High-Safety Energy Storage System and Smart Microgrids of Guangdong Province, Beijing Institute of Technology (Zhuhai), Zhuhai 519088, China; Shandong Key Laboratory of Advanced Chemical Energy Storage and Intelligent Safety, Advanced Technology Research Institute, Beijing Institute of Technology, Jinan 250300, China; Collaborative Innovation Center of Electric Vehicles in Beijing, Beijing 100081, China

**Keywords:** biological systems, Life Cells, energy acquisition, energy conversion, energy storage

## Abstract

Current battery technologies are essential for energy systems but are hindered by limitations such as low energy density, limited functionality, environmental hazards, and safety concerns, restricting their ability to meet growing global energy demands. In contrast, biological systems have evolved highly efficient, adaptive, and sustainable energy management strategies over billions of years. This review introduces ‘Life Cells’ (LCs), bio-inspired batteries designed to replicate these biological principles, enabling autonomous energy acquisition, conversion, storage, and utilization. We examine their core mechanisms, biomimetic materials, and system architectures. Furthermore, we discuss the challenges, strategies, and future opportunities for advancing LC technology and its potential applications in next-generation sustainable energy solutions.

## Teaser

Future batteries won’t just store energy, they will ‘live’. Inspired by billions of years of biological evolution, Life Cells (LCs) are a revolutionary class of batteries designed to autonomously acquire, convert, store, and utilize energy, paving the way for truly sustainable and intelligent power solutions.

## INTRODUCTION

Batteries are indispensable to modern technology, powering portable electronics, renewable energy systems, and electric transportation. However, current battery technologies, predominantly lithium-ion batteries, face significant challenges: low energy density limits device runtime, inadequate intelligence hinders dynamic adaptation, and manufacturing and disposal raise environmental concerns [1,2]. Safety issues, including overheating and leakage, further impede advancements [3]. These limitations underscore the urgent need for next-generation battery technologies that are efficient, safe, and sustainable. Living systems, honed by billions of years of evolution, provide unparalleled inspiration for addressing these challenges. Plants convert solar energy into chemical energy through photosynthesis, animals metabolize nutrients to sustain life, and certain microorganisms adapt to extreme environments via chemosynthesis. These systems exemplify not only exceptional energy efficiency but also adaptability and sustainability, traits critical for future energy solutions. Notably, both living systems and batteries rely on electron flow as a core mechanism for energy conversion, making the former an ideal model for technological innovation [4].

**Figure 1.**
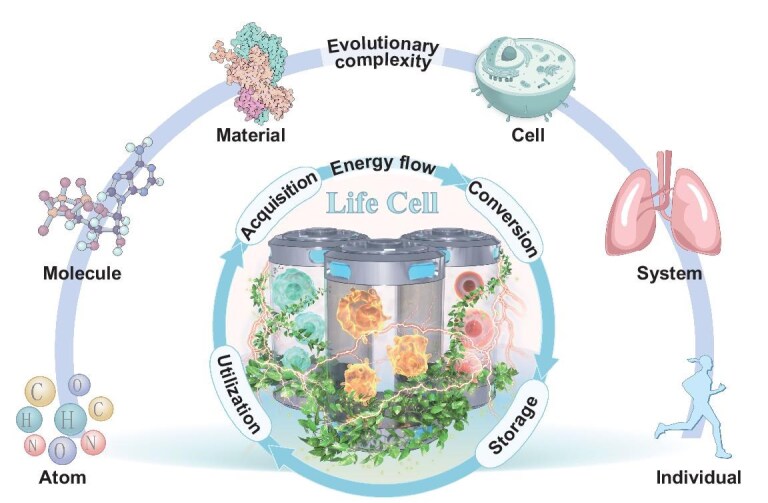
The energy flow and evolutionary process of Life Cells. LCs draw inspiration from the energy management mechanisms found in living systems, enabling the autonomous flow of energy through processes such as acquisition, conversion, storage, and utilization. By mimicking biological functions at the molecular, cellular, and systemic levels, they overcome the limitations of traditional energy storage technologies. This biomimetic approach has the potential to revolutionize energy storage, creating systems that are sustainable, efficient, and capable of adapting to changing environmental conditions.

This review introduces Life Cells (LCs), a class of life-inspired batteries that emulate the adaptive and evolutionary mechanisms of living systems (Fig. 1). LCs aim to autonomously acquire, convert, store, and utilize energy, surpassing conventional batteries in autonomy, environmental friendliness, and safety. Two primary approaches underlie LC development: harnessing electricity from biological cells through natural metabolic processes, and using biomimetic materials to replicate biological energy mechanisms for enhanced efficiency [5,6]. Modular designs further integrate multifunctional components, enabling dynamic regulation and efficient energy distribution, closely mimicking energy management in living organisms. In this review, we systematically explore the concept of LCs by clarifying their definition, distinguishing them from traditional batteries, and analysing their elemental composition, operating mechanisms, material innovations, and representative applications. We discuss technical bottlenecks and challenges, propose potential solutions, and forecast the transformative impact of LCs on emerging fields such as wearable devices, mobile energy systems, and environmental monitoring. By providing a comprehensive perspective, we aim to inspire innovative research and accelerate progress in the emerging field of life-inspired batteries.

## FROM BIOLOGICAL CELLS TO LIFE CELLS

**Figure 2.**
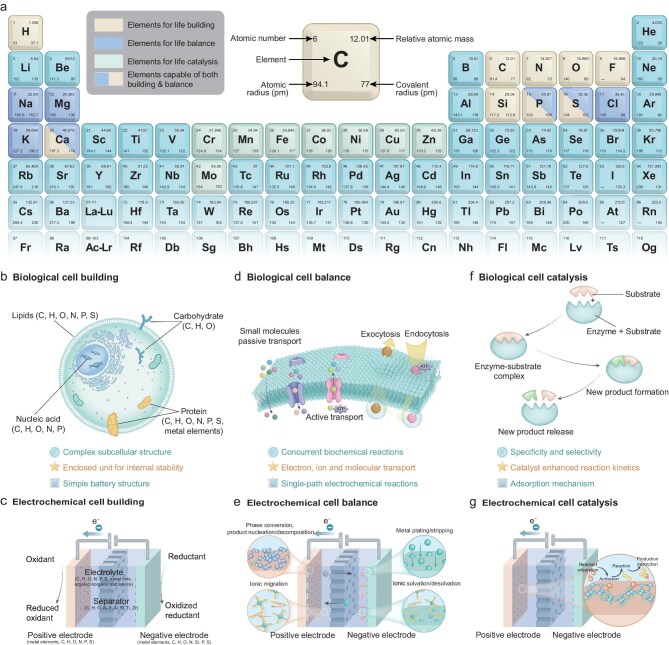
The similarities and differences between biological cells and current electrochemical cells. (a) The distribution and corresponding functions of elements selected by life in the periodic table. (b) The complex subcellular structure of cells, primarily composed of various organelles made up of carbohydrates, lipids, proteins, and nucleic acids, along with their functional compartmentalization. (c) The simple structural design of an electrochemical cell, consisting of a cathode, anode, separator, electrolyte, and an external circuit for conducting current. (d) Biological cellular balance through transmembrane transport processes, including passive transport, facilitated transport, active transport, as well as endocytosis and exocytosis. (e) Electrochemical cell balance through ion migration and electron flow, including ion transport in the electrolyte, redox reactions at the interfaces, and electron transfer in the external circuit. (f) Catalytic reactions in biological cells facilitated by enzymes. (g) Catalytic reactions in electrochemical cells facilitated by catalyst materials that adsorb reactants and promote charge transfer processes.

The term ‘cell’ holds distinct meanings across disciplines. In biology, it refers to the fundamental unit of life, while in materials science, it describes the basic unit of energy storage in batteries. Despite their differences, both biological and battery cells share core principles of energy management, encompassing acquisition, conversion, storage, and utilization. These processes rely on optimized structures, material balance, and catalytic efficiency (Fig. 2).

Life operates within the realm of chemistry. Biological systems utilize elements from the periodic table based on their abundance and accessibility. For instance, life selects C, H, O, N, P, and S to build cells and employs ions like Na^+^, K^+^, and Ca^2+^ to maintain charge balance and osmotic stability. Trace amounts of transition metals such as Mn, Fe, and Co enable redox reactions, driving efficient biochemical processes. Over billions of years, evolution has optimized these elemental cycles, ensuring sustainable reuse within ecosystems. Artificial design has expanded the range of usable elements, incorporating critical metals such as Li, Co, and Ni into advanced electrode materials to improve energy density and charge–-discharge performance. However, challenges such as resource scarcity, complex extraction processes, and low recycling efficiency hinder further development [7].

Biological and battery cells exhibit striking parallels. Both utilize relatively closed structures to maintain stability, membranes for material transport, and catalysts to accelerate reactions. Yet, significant differences remain: biological cells are dynamic, adaptive units capable of responding to environmental changes, while batteries are static devices limited to storing and releasing energy. Biological ‘life’ emerges from the integration of multiple systemic functions, including metabolism, self-regulation, environmental adaptability, information processing, and evolution. Inspired by these principles, we advance the LC concept through the embedding of life-like logic into the electrochemical system. This approach enables dynamic optimization of energy management in response to environmental feedback. By instilling this logic across material and architectural scales, we aim to transform conventional batteries from passive stores into autonomous, adaptive LCs.

## THE MULTI-ELECTRON THEORETICAL BASIS OF LIFE CELLS

LCs draw inspiration from natural energy strategies, mimicking processes like photosynthesis, respiration, and chemosynthesis. These mechanisms enable the interconversion of solar, chemical, and biomass energy with electrical energy.

Photosynthesis, as a biological method for converting solar to chemical energy, operates within the visible spectrum (400–700 nm), which accounts for ~45% of solar energy. This process comprises two stages: light-dependent reactions and the Calvin cycle. Under ideal conditions, its theoretical efficiency reaches 11%. In contrast, photovoltaic technology, governed by the Shockley–Queisser limit, achieves a maximum theoretical efficiency of 33.7% for single-junction solar cells. Advances such as perovskite materials and multijunction designs have further enhanced solar-to-electric conversion efficiency, broadening the spectrum of absorbed wavelengths [8,9].

Redox reactions form the foundation of both biological energy systems and batteries. Biological respiration and chemosynthesis use electron transfer to drive energy generation and metabolism. Similarly, batteries convert chemical energy into electrical energy through electrochemical reactions. For example, the efficiency of chemical energy conversion depends on thermodynamic parameters like Gibbs free energy (∆*G*) and enthalpy change (∆*H*), as described by Equations (1) and (2):


(1) \begin{eqnarray*}\Delta {{G}} = \Delta {{H}} - T\Delta {\mathrm{S}},\end{eqnarray*}

(2) \begin{eqnarray*}{\eta }_{(\textit{chem})} = \frac{{{E}_{ele(\textit{chem})}}}{{{E}_{\textit{chem}}}} \times 100\% \le \frac{{\Delta G}}{{\Delta H}}.\end{eqnarray*}

The decrease in Gibbs free energy determines the theoretical energy density (*E_M_*, Equation 3) and the theoretical voltage (*V*, Equation 4) of the battery, as described by the Nernst equation. Here, $\sum M$ is the total molar mass of the reactants, *n* denotes the number of electrons transferred per mole, and *F* is the Faraday constant [10,11].


(3) \begin{eqnarray*}{E}_M = \frac{{\Delta G}}{{\sum M }},\end{eqnarray*}

(4) \begin{eqnarray*}V = - \frac{{\Delta G}}{{nF}}.\end{eqnarray*}

Table 1 compares the theoretical efficiencies, voltages, and energy densities of various battery systems using oxygen as the active material of the positive electrode [12]. Light elements (e.g. Li and H) and multi-electron redox reactions are ideal for high-energy-density applications. Alkali metal negative electrodes offer higher voltages and stronger reaction driving forces. Small molecule fuels, such as alkanes and alcohols, are suitable for liquid storage. Organic materials like glucose, used as negative electrode active materials, operate under mild conditions, exhibit better biocompatibility, and have higher theoretical efficiency limits. Notably, unlike conventional batteries limited by the thermodynamic constraints of chemical reactions, LCs operate under open-system conditions where external energy inputs and internal information feedback mechanisms may contribute to system-level energy transduction. These features suggest that LCs could, in principle, exceed the theoretical efficiency limits defined for closed chemical systems.

**Table 1.** Theoretical parameter comparison of batteries using oxygen as the positive electrode active material under standard conditions.


		△*G*	△*H*	Energy density	Voltage	Efficiency
Reaction (H_2_O refers to liquid water)	Number of electrons	(kJ/mol)	(kJ/mol)	(kWh/kg)	(V)	(%)

2Li + O_2_ → Li_2_O_2_	2 e^−^**/**mol O_2_	−571.1	−634.3	3.46	2.96	90.0
4Li + O_2_ → 2Li_2_O	4 e^−^**/**mol O_2_	−1122.4	−1195.8	5.21	2.91	93.9
Na + O_2_ → NaO_2_	1 e^−^**/**mol O_2_	−218.4	−260.2	1.11	2.27	83.9
K + O_2_ → KO_2_	1 e^−^**/**mol O_2_	−239.4	−284.9	0.93	2.48	84.1
2Zn + O_2_ → 2ZnO	4 e^−^**/**mol O_2_	−641.0	−701.0	1.35	1.65	91.4
2H_2_ + O_2_ → 2H_2_O	4 e^−^**/**mol O_2_	−474.4	−571.7	3.67	1.23	83.0
2/3CH_3_OH + O_2_ → 2/3CO_2_ + 4/3H_2_O	4 e^−^**/**mol O_2_	−467.9	−484.0	2.44	1.21	96.7
1/2CH_4_ + O_2_ → 1/2CO_2_ + H_2_O	4 e^−^**/**mol O_2_	−409.1	−445.3	2.84	1.06	91.9
2/7C_2_H_6_ + O_2_ → 4/7CO_2_ + 6/7H_2_O	4 e^−^**/**mol O_2_	−419.5	−446.9	2.87	1.09	93.9
1/3C_2_H_5_OH + O_2_ → 2/3CO_2_ + H_2_O	4 e^−^**/**mol O_2_	−441.7	−455.6	2.59	1.14	96.9
1/6C_6_H_12_O_6_ + O_2_ → CO_2_ + H_2_O	4 e^−^**/**mol O_2_	−479.8	−467.1	2.15	1.24	102.7^a^

a The calculated theoretical conversion efficiency of biofuel cells using glucose as fuel slightly exceeds 100% due to experimental approximations and measurement errors.

**Figure 3.**
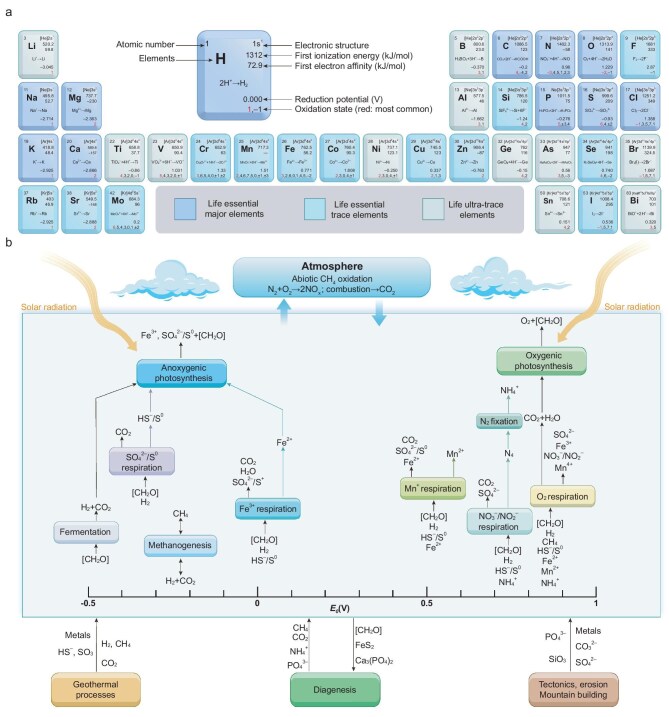
Typical electrode potentials of life elements and their roles in natural energy flow. (a) Typical chemical reduction reactions involving life elements, reduction potentials referenced to the SHE. (b) Energy and matter cycles driven by biochemical processes: the cycling of six key elements that constitute biological macromolecules (H, C, N, O, S, and P) on Earth is primarily driven by biologically catalyzed, thermodynamically constrained redox reactions. Photosynthesis, for example, relies on light energy penetrating the atmosphere to oxidize electron donors (such as H_2_O, HS^−^, H_2_, or Fe^2+^). The resulting electrons and protons are used to reduce inorganic carbon into organic compounds, forming high-energy bonds for biological utilization. Concurrently, geological processes such as volcanic activity and rock weathering recycle buried elements like carbon, sulfur, and phosphorus back to the surface, sustaining their availability for life. Panel b is adapted from Ref.^ 13^, American Association for the Advancement of Science.

As shown in Fig. 3a, the reduction potentials of various life elemental substances and major compounds can guide the design of advanced LCs [13]. These insights into natural and engineered systems lay the groundwork for constructing LCs that emulate biological energy mechanisms. In Fig. 3b, the dominant redox reactions in biological systems are distributed mainly within the potential window of −0.5 to 1.0 V (versus the standard hydrogen electrode, SHE). This electrochemical window reflects an evolutionary optimization that balances energy efficiency and system stability. Larger redox gaps could yield higher energy outputs but often trigger undesirable side reactions, irreversible structural changes, or the generation of reactive oxygen species (ROS), while smaller gaps reduce energy utilization and impair critical biochemical pathways. This trade-off represents a form of natural electrochemical homeostasis, tightly coupled with the biogeochemical cycling of life-essential elements such as C, N, S, and Fe. Inspired by such natural designs, the development of LCs could follow similar principles, employing renewable redox couples within this biologically relevant voltage range to ensure inherent system robustness and long-term cycling stability.

However, unlike biological systems constrained by enzymatic limits and aqueous stability, LCs are not strictly bound by these evolutionary restrictions. Through artificial modulation strategies, such as tuning ionic activity, modifying solvation shells, or engineering interfacial structures, it becomes possible to expand the accessible redox range while maintaining self-regulation, adaptability, and dynamic control [14–16]. These capabilities allow LCs to retain life-like energy behaviours across a broader electrochemical landscape, thus combining biological inspiration with engineering flexibility to meet specific energy conversion and functional needs.

## ENERGY SOURCES AND CONVERSION MECHANISMS OF LIFE CELLS

**Figure 4.**
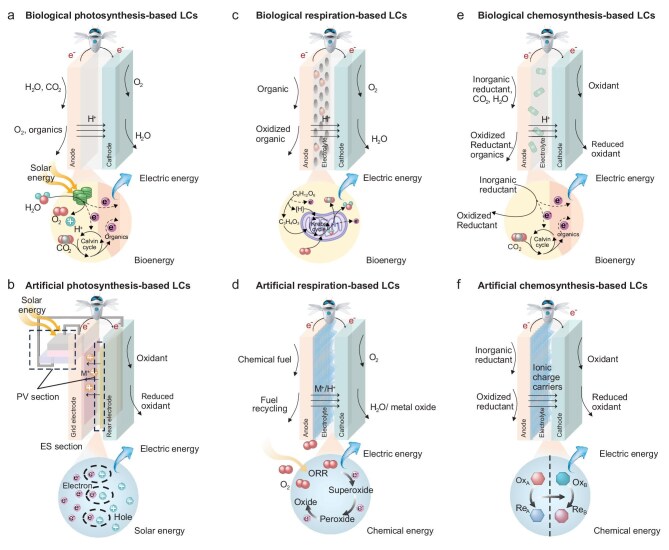
Operating mechanisms of LCs inspired by biological cells. (a) Photosynthesis-based LCs extract electrons from the light or dark reactions of photosynthetic cells to the negative electrode. The electrons then transfer through an external circuit to the positive electrode. (b) Artificial photosynthesis-based LCs absorb solar energy through a light-harvesting active layer, generating electrons and holes. These charges are separated by the built-in electric field within the active layer and then collected and transported via electron and hole transport layers. (c) Respiration-based LCs collect electrons generated during the decomposition of organic substances through cellular respiration at the negative electrode. These electrons are then transferred to the positive electrode, where oxygen is reduced. (d) Artificial respiration-based LCs extract electrons generated during the oxidation of fuel or active metals at the negative electrode. These electrons are transferred to the positive electrode to reduce oxygen. (e) Biological chemosynthesis-based LCs extract electrons during the oxidation of inorganic substances by chemosynthetic autotrophic bacteria. These electrons are then transferred to reduce oxidizing agents. (f) Artificial chemosynthesis-based LCs generate electrons by oxidizing *in-situ* acquired inorganic substances (e.g. H_2_, H_2_S) at the negative electrode, and consume electrons at the positive electrode through the reduction of oxidizing agents.

LCs draw inspiration from the energy acquisition strategies of living organisms, categorized into three main types: photosynthesis-based, respiration-based, and chemosynthesis-based LCs (Fig. 4). Each type includes biological LCs, which leverage living cells or their products, and artificial LCs, which mimic biological energy management mechanisms.

### Photosynthesis-based Life Cells

Green plants convert carbon dioxide and water into carbohydrates and oxygen through photosynthesis, sustaining the Earth’s carbon-oxygen cycle. In chloroplasts, light-harvesting pigments absorb specific wavelengths to split water into oxygen, electrons, and protons. These processes store solar energy as adenosine triphosphate (ATP) and reduced nicotinamide adenine dinucleotide phosphate (NADPH), which drive the Calvin cycle to stabilize energy in carbohydrates. Photosynthesis-based LCs replicate this mechanism by extracting electrons from photosystems or mimicking photosynthetic pathways to generate electricity.

### Biological photosynthesis-based Life Cells

Excited-state electrons generated during natural photosynthesis can be directly extracted from the photosystems or indirectly extracted via oxidation reactions from reducing carriers such as plastoquinone, NADPH, or carbon fixation products. Once these electrons are released from the biological cells, they can enter a bioelectrochemical system to drive a biological photovoltaic process (Fig. 4a) [17]. In this process, electrons are received by the negative electrode and conducted through an external circuit to the positive electrode to generate current [18,19]. For instance, a biological photovoltaic system utilizing an aluminium negative electrode coated with the photosynthetic bacterium Synechocystis sp. PCC6803 has been shown to continuously power a microprocessor for over 6 months [20].

### Artificial photosynthesis-based Life Cells

Inspired by natural photosynthesis, artificial systems integrate photovoltaic (PV) and electrochemical storage (ES) components to enhance energy conversion and storage (Fig. 4b) [21]. The photovoltaic part consists of a light-harvesting layer, an electron transport layer, and a hole transport layer. After light is absorbed in the light-harvesting layer, the generated electrons and holes move in opposite directions under the influence of the built-in electric field. These charges reach the electrodes via the transport layers, creating a potential difference and generating an electric current [22]. However, challenges like intermittent sunlight and power transmission persist [23]. Integrating light-harvesting and energy storage within photoresponsive electrodes offers a promising dual-functional approach, improving cycling efficiency and reducing overpotential [24].

### Respiration-based Life Cells

Cellular respiration oxidizes organic compounds to release energy through enzymatic reactions. Aerobic respiration consists of glycolysis, the Krebs cycle, and the electron transport chain, culminating in ATP synthesis and water production. Respiration-based LCs mimic these processes to convert chemical energy into electricity, using either biological catalysts or biomimetic systems.

### Biological respiration-based Life Cells

Biofuel cells use biocatalysts like enzymes or microorganisms to oxidize organic fuels, generating electricity (Fig. 4c) [25]. Enzymatic biofuel cells excel in catalytic efficiency and specificity but face challenges like enzyme stability and cost [26]. Microbial fuel cells (MFCs) utilize active microorganisms for extracellular electron transfer, with applications in wearable devices and flexible electronics. The electrons released during metabolism transfer either directly or indirectly to the negative electrode. Direct transfer relies on nanowires or conductive pigment proteins, while indirect transfer uses redox mediators. However, the low efficiency of extracellular electron transfer remains a significant challenge for the application of MFCs [27].

### Artificial respiration-based Life Cells

Fuel cells and metal-air batteries replicate respiration by using oxygen as the electron acceptor (Fig. 4d). For example, hydrogen-oxygen fuel cells achieve high efficiency with water as the sole product [28], while lithium-air batteries achieve energy densities up to 3500 Wh/kg, surpassing traditional batteries. However, in practical applications, kinetic limitations and side reactions lead to poor rate performance and cycling stability. Future advancements in biomimetic catalysts and electrolyte designs aim to overcome kinetic and stability limitations, unlocking their potential for sustainable energy solutions [29]. Moreover, natural electron-carrying cofactors such as heme, coenzyme Q, and vitamin K can significantly improve oxygen reduction reaction performance [30].

### Chemosynthesis-based Life Cells

Chemosynthesis-based LCs draw inspiration from microorganisms that thrive in extreme environments by extracting electrons from inorganic compounds. Unlike respiration-based LCs, these systems use inorganic electron donors. Chemosynthetic microorganisms, including nitrifying bacteria and sulphur bacteria, sustain life by oxidizing specific inorganic compounds, even under extreme conditions like high temperatures, pressures, or acidity. This energy utilization strategy offers insights for designing advanced LCs suitable for harsh environments.

### Biological chemosynthesis-based Life Cells

Electroactive chemosynthetic microorganisms can transfer electrons externally during metabolic processes, enabling direct energy harvesting for LCs (Fig. 4e). For instance, *Geobacter sulfurreducens* produces protein nanowires that generate voltage (~0.5 V) and current (~17 μA/cm^2^) under humidity gradients [31]. These biofilms exhibit power densities of ~1 μW/cm^2^, sufficient for low-power wearable devices [32]. Similarly, *Rhodopseudomonas palustris* biofilms generate continuous voltage (0.29–0.34 V) and current (0.25–0.50 μA) for at least 50 days [33]. However, challenges such as limited extracellular electron transfer, long growth cycles, and stringent environmental requirements hinder their large-scale applications [34]. Nonetheless, the adaptability of extremophiles like hyperthermophilic microorganisms in volcanic craters or hydrothermal vents inspire novel LCs for extreme environments [35].

### Artificial chemosynthesis-based Life Cells

Artificial chemosynthesis-based LCs mimic the metabolic pathways of chemosynthetic microorganisms to convert chemical energy into electricity (Fig. 4f). These systems use functionalized electrodes and biomimetic catalysts to capture electrons from inorganic compounds, such as sulfides, Fe^2+^, or H_2_, and reduce electron acceptors like O_2_, N_2_, or CO_2_. Such designs enable *in-situ* energy utilization in extreme environments. For example, a Li-CO_2_ battery designed for the Martian atmosphere achieved an energy density of 373.9 Wh/kg and a cycle life of 1375 hours at 0°C, demonstrating the feasibility of energy storage for Martian exploration [36].

## BIOMIMETIC FUNCTIONAL MATERIALS FOR LIFE CELLS

**Figure 5.**
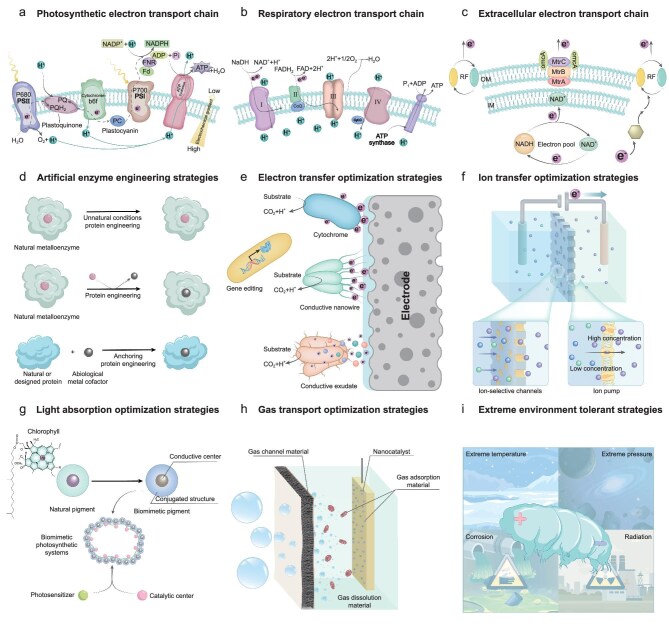
Functional materials and biomimetic optimization strategies for LCs. (a) Photosynthetic electron transport chain: photosynthetic pigments capture light energy, transferring high-energy electrons to the reaction centre, where water is split into O_2_ and H^+^. Electron transport across the thylakoid membrane generates NADPH and drives H^+^ flow through ATP synthase, synthesizing ATP. (b) Respiratory electron transport chain: in aerobic respiration, electrons from reduced nicotinamide adenine dinucleotide (NADH) are transferred through proteins in the mitochondrial inner membrane, combining with O_2_ and H^+^ to form H_2_O. This electron flow creates an H^+^ electrochemical gradient, driving ATP synthesis via ATP synthase. (c) Extracellular electron transport chain: electroactive microorganisms transfer electrons generated by intracellular metabolism to external acceptors via outer membrane cytochromes, conductive pili, and electron shuttle molecules, optimizing extracellular electron flow. (d) High-activity artificial enzyme engineering strategies: enhancing catalytic activity by replacing or embedding metal cofactors into protein scaffolds. (e) Electron transfer optimization strategies: improving electron transfer efficiency using conductive materials or optimized electron mediators. (f) Biomimetic ion-selective channels and ion pumps enhance ion selectivity and ion transport efficiency. (g) Light absorption materials mimic natural pigments and photosynthetic complexes to achieve efficient energy conversion. (h) Gas transport materials. (i) Extreme environment tolerant materials mimic biological defence mechanisms.

Efficient energy conversion in LCs hinges on optimizing electron transport chains, the core pathway for energy flow. In living systems, electron transport chains consist of carriers ordered by electron affinity and are embedded in membranes like the mitochondrial inner membrane or chloroplast thylakoids. These chains drive ATP synthesis via proton gradients (Fig. 5a and 5b) [37,38]. In electroactive microorganisms, extracellular electron transport chains exhibit greater adaptability, transferring electrons to external acceptors like Fe^3+^ or electrodes through cytochromes, pili, or mediators (Fig. 5c) [39]. Biomimetic materials replicate these mechanisms to enhance energy harvesting and conversion while overcoming limitations in conventional materials.

### Catalytic materials

Catalytic materials reduce activation energy in all LC types. Natural enzymes achieve high efficiency through microenvironments formed by amino acids and cofactors (mostly metal ions) [40], stabilizing substrates’ transition states via molecular interactions such as hydrogen bonding, hydrophobic interactions, and van der Waals forces [41]. Mimicking these mechanisms, nanozymes (including metal nanoparticles, metal oxides, and carbon nanomaterials) offer high activity and cost-effectiveness but face challenges in substrate specificity and biocompatibility.

Protein-based enzymes, optimized through genetic engineering and rational design, enable multi-step reactions and exhibit greater flexibility. For instance, artificial protein-metal enzymes embed metal cofactors into protein scaffolds, imparting novel functionalities (Fig. 5d) [42,43]. Multi-enzyme cascade systems, inspired by Photosystems I-II and respiratory chain complexes I-IV, maximize substrate utilization, enhancing LC energy density and output [44,45]. In the future, advancements in artificial intelligence predictions, protein engineering, and synthetic biology are expected to enable the design of high-performance biological enzyme catalysts, driving more efficient energy conversion in LCs [46,47].

### Electron transfer materials

The electron transfer materials in LCs are categorized into endogenous, exogenous, and electrode materials. Optimizing these components and their interactions significantly enhances energy conversion efficiency and broadens LCs application potential (Fig. 5e). Endogenous electron transfer materials include cytochromes, conductive pili (nanowires), and conductive exudates like flavonoids and NADPH [48]. These materials, naturally occurring in biological cells, facilitate electron transfer but are limited in efficiency.

To overcome these limitations, exogenous materials such as graphene, metal nanoparticles, and conductive polymers are used to modify electrode interfaces, increasing surface area and active sites for enhanced electron collection [17,49]. Soluble mediators, like quinones and ferrocyanides, further facilitate electron transfer but face challenges such as cytotoxicity, high cost, and low stability [50]. Biomimetic designs and heterologously expressed cytochromes or nanowires improve these pathways, advancing LC performance [51].

Biocompatible and eco-friendly electrode materials are essential for the sustainability of LCs. Biochar, with its availability, low cost, and environmental friendliness, is a promising candidate [52]. Its tuneable porous structure and functionalized surface chemistry support efficient gas transport, ion diffusion, and electron transfer. For example, macropores enhance gas transport, micropores increase active sites, and oxygen-functional groups boost electron transfer. Adjusting pyrolysis parameters enables precise engineering of biochar to meet the electrochemical requirements of LCs [53]. This flexible design enables biochar materials to significantly enhance energy conversion efficiency and long-term stability.

### Ion transport materials

Ion transport materials are essential for maintaining ion balance in LCs and play a pivotal role in energy transfer and conversion. While conventional batteries emphasize ionic conductivity, stability, and broad temperature applicability, LCs require materials that combine high selectivity, biocompatibility, and dynamic regulatory capabilities to meet the demands of complex biological environments [54]. In biological systems, ion channels and pumps, such as proton pumps, calcium channels, and sodium-potassium pumps, regulate ion transport across membranes via electrochemical gradients or external stimuli like light, electrical signals, or chemical changes. These mechanisms not only ensure precise ion control but also maintain system stability under dynamic conditions.

Inspired by these natural systems, biomimetic ion channels and pumps have shown significant potential for LC applications (Fig. 5f) [55]. For example, graphene oxide–based biomimetic ion channels achieve precise ion selection and efficient transport through nanopore size control and surface chemical modifications [56,57]. Similarly, biomimetic ion pumps, capable of active ion transport without external power, enhance ion balance and overall system stability [58]. Moreover, multifunctional biomimetic separators, such as cellulose nanofibers, combine high selectivity, mechanical stability, and environmental adaptability. When combined with traditional separators (e.g. embedding biomimetic nanotubes or active ion pumps), they enable adaptive ion control, improving the overall energy output and lifespan of LCs [59,60].

### Light absorption materials

In photosynthesis-based LCs, light absorption materials include natural pigments and biomimetic pigments. Natural pigments such as chlorophyll, anthocyanins, and carotenoids exhibit excellent light absorption properties due to their conjugated systems (e.g. benzene rings, polyene chains, porphyrin rings) and functional groups (e.g. hydrocarbon, carboxyl, carbonyl) [61–63]. They are cost-effective, biodegradable, and environmentally friendly. In dye-sensitized solar cells, these pigments efficiently absorb visible and near-infrared light, maintaining stable energy output even under low-light conditions. However, challenges such as low power conversion efficiency (2%–5%) and instability to light, heat, and pH changes limit their direct application [64].

Biomimetic pigments enhance the stability and absorption range of natural pigments (Fig. 5g). Chemical modifications reduce molecular band gaps, broadening the absorption spectrum [65]. Artificial photosensitizers, using metal complexes like ruthenium or iridium, mimic chlorophyll’s electron transfer properties [66]. Inspired by natural photosynthetic systems, the light-to-electricity conversion efficiency can be enhanced to 4.2% through multi-pigment complexes and biomimetic chlorophyll-derived multilayer structures [67,68]. Techniques such as supramolecular self-assembly further integrate light-absorbing and catalytic materials, improving charge transfer and energy utilization [69,70].

### Gas transport materials

In respiration-based LCs, efficient gas transport and dissolution (e.g. O_2_) and timely removal of intermediate products (e.g. CO_2_) are critical for maintaining high battery performance. Gas transport materials include gas channel materials, gas dissolution materials, and gas adsorption materials (Fig. 5h). Efficient biological gas exchange channels, such as alveolar walls and gill filaments, inspire the design of materials like porous carbon paper, carbon cloth, and metal-organic frameworks. These materials enhance gas transport uniformity and efficiency by precisely controlling pore size and surface properties [71]. Gas dissolution materials improve the solubility of O_2_ and CO_2_ in electrolytes, mimicking blood’s dynamic gas-handling capabilities. Optimizing electrolyte composition and its interaction with porous materials accelerates gas transfer and dissolution, ensuring continuous energy conversion [72].

Haemoglobin’s ability to dynamically regulate O_2_ and CO_2_ based on partial pressure differences inspires the development of gas adsorption materials tailored to varying gas demands during LC operation [73,74]. Integrating covalent organic frameworks with Nafion to construct dynamic water-locking ion conductors, inspired by thermophilic bacteria, reduces water vapour pressure at high temperatures (105°C), enhancing oxygen transport and increasing fuel cell power density by 1.9 times [75]. Biomimetic nanoparticles, such as platinum-based catalysts, further improve adsorption/desorption efficiency and accelerate reaction kinetics.

### Extreme environment tolerant materials

Chemosynthesis-based LCs demand materials that withstand extreme conditions such as high temperatures, pressure, corrosive media, and radiation, which can degrade atomic bonding and microstructure. Developing high-performance materials capable of enduring such environments is a critical research focus. Natural adaptations in polar organisms, deep-sea microbes, and halophiles provide valuable insights for designing resilient materials for chemosynthesis-based LCs (Fig. 5i) [76]. For instance, polar organisms’ membrane lipids maintain flexibility at low temperatures, inspiring low-temperature adaptive materials. Deep-sea microorganisms rely on protein cooperation for high-pressure resistance, offering strategies for robust structural designs [77]. Halophilic microbes adapt to high-salt conditions through unique ion-regulating mechanisms, while acidophiles’ proteins, enriched with acidic amino acids and hydrophobic residues, inform the development of salt- and acid-resistant materials [78].

Derived biomimetic strategies include mimicking mussel foot proteins to create highly adhesive, corrosion-resistant coatings, extending battery lifespans under strong acidic or alkaline conditions [79]. Dynamic regulation materials, inspired by soil-microorganism interactions, adapt surface chemistry and ion exchange capacity to enhance electrochemical performance and self-healing abilities [80]. Low-temperature biomimetic materials, incorporating phenylpolydimethylsiloxane elastomers and microfibril structures, demonstrate high-strength and reversible adhesion properties [81]. Additionally, combining the protein assembly traits of deep-sea microorganisms with acidophiles’ adaptations enables the creation of high-pressure and acid-resistant materials [82].

As representative systems of endogenous intelligent energy, LCs are fundamentally supported by a suite of synergistic functional materials. Inspired by biological electron transport chains, ion channels, and adaptive regulatory pathways, these materials must not only exhibit excellent electrochemical properties, but also demonstrate adaptive responsiveness, perturbation resilience, and functional plasticity. Such integration lays the foundation for a mechanism-material-function coupling paradigm that defines the architectural logic of LCs.

## SOPHISTICATED SYSTEMS FOR LIFE CELLS

**Figure 6.**
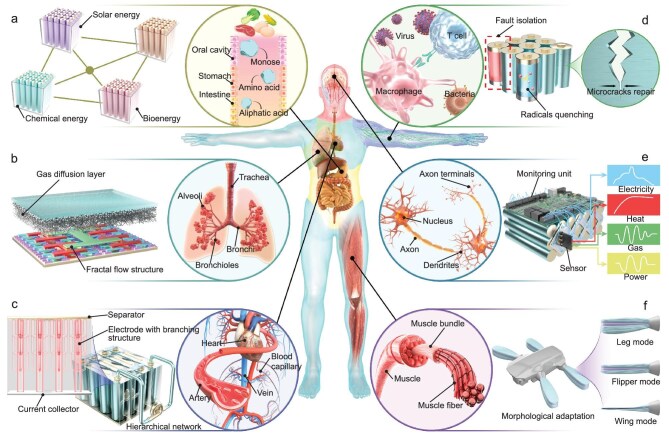
Sophisticated LC systems inspired by the six major human systems. (a) Multi-energy input module inspired by the digestive system. (b) Oxidation-mediated module inspired by the respiratory system. (c) Energy-matter transfer network inspired by the circulatory system. (d) Self-healing module inspired by the immune system. (e) Monitoring and feedback module inspired by neuro system. (f) Dynamic adaptation module inspired by muscle system.

In future energy systems, single LC units often struggle to meet complex energy demands due to limited functionality and adaptability, much like single-celled organisms. Overcoming these limitations requires multi-unit integration, where advanced systems, inspired by multifunctional organs and systems in the human body, enable efficient management and adaptive regulation of energy networks (Fig. 6).

### Multi-energy input module inspired by the digestive system

The human digestive system efficiently converts diverse energy sources into absorbable forms through biochemical processes, inspiring multi-energy input modules for LCs. These modules integrate solar, biomass, and chemical energy through modular and intelligent management, ensuring efficient electrical energy conversion and distribution [83]. AI- and machine learning–based scheduling algorithms monitor energy node status in real-time, predict future demands, and adjust conversion paths, ensuring reliable and efficient operation of LC systems in multi-energy input scenarios [84].

### Oxidation-mediated module inspired by the respiratory system

The respiratory system delivers oxygen from air to drive cellular oxidation, inspiring oxidation-mediated modules in LCs. These modules mimic the lung’s fractal flow structure, enabling distributed gas delivery and multi-point exchange [85,86]. This optimized flow system significantly enhances oxidant utilization efficiency and the stability of gas exchange [87]. Integrated sensors monitor oxidation conditions of LCs, dynamically optimizing reactions to maximize energy output, akin to the oxygen balance in biological respiration [88,89].

### Energy-matter transfer network inspired by the circulatory system

The circulatory system efficiently distributes oxygen and nutrients through a hierarchical network, offering a blueprint for LC multi-level energy-matter transfer systems [64,90]. Mimicking this branching architecture accelerates reactant transfer and reduces polarization. Artificial neural networks can be further employed to rapidly and accurately compute potential structures, facilitating the optimization of vascular parameters and preventing single-point failures, enhancing system reliability and safety [91].

### Self-healing module inspired by the immune system

The immune system’s fault detection and repair mechanisms inspire self-healing modules in LCs. These modules detect and isolate faulty cells, leveraging redundancy to ensure uninterrupted operation [92]. Inspired by self-repair processes, bio-inspired materials and active repair algorithms restore microcracks in battery units, extending cycle life and reducing system downtime [93,94]. Reactive oxygen species are vital antimicrobial agents and signalling molecules but can harm living systems in excess. Bioinspired strategies, such as introducing natural antioxidants or cerium oxide nanozymes with catalase-like activities, effectively mitigate excessive ROS, ensuring LC system stability [95,96].

### Monitoring and feedback module inspired by neuro system

Real-time monitoring and feedback in the nervous system inspire energy management in LC systems. Distributed built-in sensors, akin to sensory receptors, track energy inputs and outputs, supporting efficient responses to dynamic environments [94]. These sensor nodes monitor electrical, thermal, gas, and mechanical signals, relaying data to central controllers to stabilize energy flow and optimize distribution [97]. Building on the sensor network’s data acquisition capabilities, machine learning algorithms enable adaptive energy control, mimicking synaptic learning mechanisms in the nervous system [98,99]. Deep learning techniques further enhance this process by predicting energy demand fluctuations and dynamically adjusting strategies to suit [100].

### Dynamic adaptation module inspired by the muscle system

The muscle system’s ability to dynamically adjust output to external demands inspires adaptive LC modules. These modules mimic muscle responsiveness, using sensors to monitor battery status and load, reallocating energy in real time to optimize performance. This ensures efficiency and stability in applications like robotics, electric vehicles, and wearables, while extending battery life [101]. Flexible materials and real-time control algorithms further enhance adaptability, enabling morphological adjustments for efficient operation in complex environments [102]. This dynamic approach extends LC applications to aerospace, marine engineering, and other advanced energy systems, driving innovation across diverse fields.

## CONCLUSIONS AND OUTLOOK

**Figure 7.**
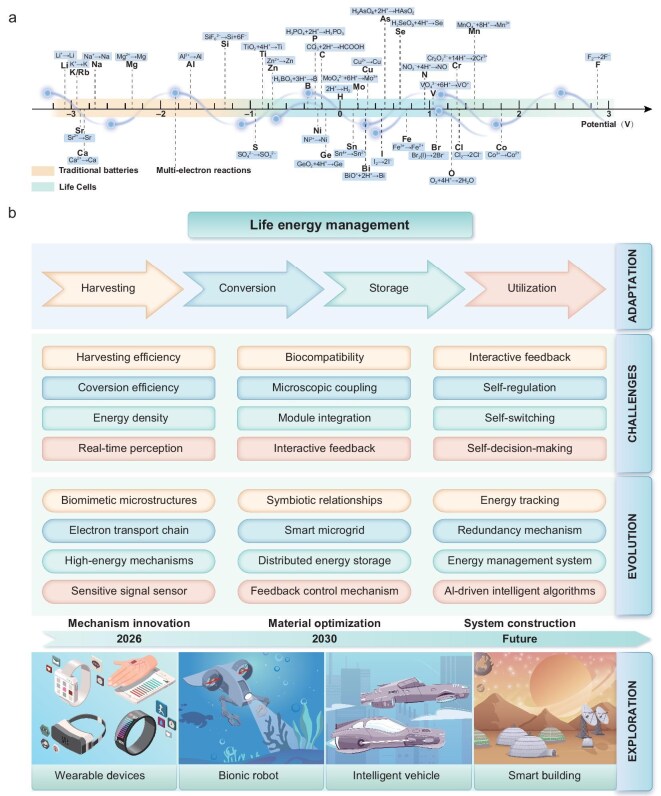
Technological roadmap for the future development of LCs. (a) Candidate multi-electron redox reactions for LCs. (b) Mapping of challenges and evolutionary pathways in the life energy management of LCs.

The development of LCs presents both significant challenges and transformative opportunities. This cutting-edge field demands not only a deep understanding of natural principles but also continuous innovation across mechanisms, materials, and systems. LCs adopt and simulate biomimetic multi-electron redox reactions in living organisms (Fig. 7a), offering a path toward efficient and sustainable energy storage. Through rational electrolyte design and electrochemical modulation, these systems have the potential not only to emulate natural redox processes but also to surpass the intrinsic 1.5 V biological redox window, unlocking new redox couples, broader potential ranges, and transformative applications beyond traditional batteries. This section explores the key challenges, potential solutions, and broader implications for future energy systems (Fig. 7b).

### Mechanism innovation

Drawing inspiration from biological energy processes, LC systems aim to integrate multiple energy sources (chemical, light, and biomass), while optimizing conversion pathways. Nature’s metabolic pathways, such as ATP synthesis driven by ion gradients or electric eel–inspired bioelectricity generation, provide valuable blueprints for enhancing energy efficiency and stability [103]. Innovations like light-electrochemical coupling in LCs can enable solar energy conversion during the day and chemical energy conversion at night, offering efficient solutions for fluctuating energy demands.

### Materials optimization

Efficient, sustainable materials are essential for the long-term viability of LCs. Biomimetic catalysts, inspired by enzyme functions, enhance energy conversion through artificial enzyme mimicry and cascade catalysis. Electron transfer efficiency can be improved with advanced materials like carbon nanotubes, graphene, and molecular wires. Ion transport materials, such as biomimetic ion channels and pumps, can balance high ionic conductivity with biocompatibility. Machine learning and high-throughput screening further accelerate the discovery of green energy materials, enabling a deeper understanding of structure–activity relationships and opening new possibilities for energy storage and conversion.

### System construction

Inspired by the multi-level coordination of biological systems, LC systems require collaborative modules to maintain dynamic equilibrium. Biological systems like the respiratory, circulatory, and nervous systems provide models for efficient energy management. Future LC designs will incorporate smart sensors, machine learning, and big data analytics to enable predictive optimization, dynamic energy allocation, and robust operation in complex environments. This intelligent, multi-module approach ensures efficiency and adaptability in LC systems.

### Future applications

LC systems hold immense promise across civilian, military, and exploratory applications. In the civilian sector, miniaturized devices such as electronic skin, soft robots, and artificial organs powered by LCs could transform healthcare, education, and human–machine interactions. In defence, lightweight and flexible LC systems provide reliable power for rapid-response equipment. In synthetic ecosystems or space exploration, LCs offer autonomy and adaptability to cope with extreme environments. Looking ahead, LCs may emulate biological energy metabolism by harnessing mechanisms such as glucose oxidation or light-driven redox cycles to enable self-sustained, nutrient-based energy harvesting. Inspired by biological aging and regeneration, future LCs could also incorporate programmed degradation and self-repair, improving lifecycle management. Together, these features position LCs as foundational components for energy-autonomous platforms such as soft robotics and bio-integrated electronics.

### Regulatory and safety concerns

Despite their promise, the path from laboratory innovation to widespread LC deployment is fraught with regulatory, safety, and ethical challenges. The complexity of LC materials and their dynamic, coupled mechanisms introduces nonlinear behaviours and system uncertainties that currently lack comprehensive safety assessment frameworks. Moreover, LCs’ convergence of energy and information blurs the traditional boundaries between batteries, electronic devices, and bioinspired materials, rendering existing regulatory classifications inadequate. Particularly in scenarios involving implantable systems, brain-machine interfaces, and autonomous human-machine integration, LCs’ potential for adaptive learning and evolution raises issues of privacy, ethics, and data sovereignty. Therefore, a unified standard system encompassing materials, devices, systems, algorithms, and ethics is urgently needed. This framework must address material biocompatibility, algorithmic explainability, regulatory compliance, and real-world deployment safety to support the responsible development of LC technologies.
